# Developmental Exposure to Ethinylestradiol Affects Reproductive Physiology, the GnRH Neuroendocrine Network and Behaviors in Female Mouse

**DOI:** 10.3389/fnins.2015.00463

**Published:** 2015-12-09

**Authors:** Lyes Derouiche, Matthieu Keller, Mariangela Martini, Anne H. Duittoz, Delphine Pillon

**Affiliations:** PRC, UMR 7247 INRA/CNRS/Université François-Rabelais de Tours/IFCENouzilly, France

**Keywords:** ethinylestradiol, reproduction, GnRH, neuroendocrinology, sexual behavior, endocrine disruption

## Abstract

During development, environmental estrogens are able to induce an estrogen mimetic action that may interfere with endocrine and neuroendocrine systems. The present study investigated the effects on the reproductive function in female mice following developmental exposure to pharmaceutical ethinylestradiol (EE2), the most widespread and potent synthetic steroid present in aquatic environments. EE2 was administrated in drinking water at environmentally relevant (ENVIR) or pharmacological (PHARMACO) doses [0.1 and 1 μg/kg (body weight)/day respectively], from embryonic day 10 until postnatal day 40. Our results show that both groups of EE2-exposed females had advanced vaginal opening and shorter estrus cycles, but a normal fertility rate compared to CONTROL females. The hypothalamic population of GnRH neurons was affected by EE2 exposure with a significant increase in the number of perikarya in the preoptic area of the PHARMACO group and a modification in their distribution in the ENVIR group, both associated with a marked decrease in GnRH fibers immunoreactivity in the median eminence. In EE2-exposed females, behavioral tests highlighted a disturbed maternal behavior, a higher lordosis response, a lack of discrimination between gonad-intact and castrated males in sexually experienced females, and an increased anxiety-related behavior. Altogether, these results put emphasis on the high sensitivity of sexually dimorphic behaviors and neuroendocrine circuits to disruptive effects of EDCs.

## Introduction

Evidence that exposure to Endocrine Disrupting Chemicals (EDCs) during development contributes to disturbing various parameters of animal and human reproductive function, such as puberty onset, fertility, and behaviors, has been largely highlighted (Walker et al., 2014; Parent et al., 2015). It is widely accepted that natural hormones and potentially EDCs modulate the development of the central and peripheral nervous systems, including the setting of neuroendocrine circuits controlling physiological and behavioral outcomes of reproductive function (McCarthy, 2008; Gore et al., 2011). In mammals, hypothalamic neuroendocrine circuits orchestrating the pituitary-gonadal activity are established during prenatal, early postnatal, and juvenile periods under the organizational effects of specific patterns of endogenous estrogens, leading to masculinization or feminization of a bipotential developing brain (McCarthy, 2008). In male, late fetal and postnatal testosterone aromatized to estradiol is responsible for neuroanatomical and functional masculinization of neuroendocrine circuits, leading to the expression of adult male typical sexual behaviors (Bakker, 2003). In female, perinatal brain develops in much lower steroid hormones and is protected from maternal estrogens by the alpha-fetoprotein, which binds estradiol with high affinity (Bakker et al., 2006). Thus, disruption of the ongoing patterns of estrogens during development may durably alter the establishment of neuroendocrine networks and consequently affect physiological, neuroendocrine and behavioral components of reproductive function in adulthood.

Pharmaceutical 17α-ethinylestradiol (EE2) is a potent estrogenic compound that is used mainly in oral contraceptives. EE2 is among the most dominant environmental estrogens (Snyder et al., 2001; Pojana et al., 2004; Laurenson et al., 2014). Its concentration in aquatic environments is highly variable according to environmental localization throughout the world. In the USA and Europe, EE2 has been detected in surface water at concentrations ranging from non-detectable to 273 ng.L^−1^ (Pojana et al., 2004; Hannah et al., 2009). In Asia, EE2 concentrations are largely higher, reaching 4100 ng.L^−1^ in some wastewater treatment plants (WTPs) in Beijing (Zhou et al., 2012). Moreover, a low rate of EE2 removal from wastewater (20%) may considerably contribute to its bioaccumulation in WTP outputs and potentially in natural aquatic environments (Ternes et al., 1999; Balsiger et al., 2010). Given the concern raised by the large EE2 pollution, it has thus received increasing attention and, recently, the European Parliament and the Council of Europe added EE2 to the priority “Watch list” of substances presenting a significant risk to or *via* aquatic environments according to the Environmental Quality Standards (Directive 2013/39/EU)^1^. Due to its high estrogenic potency and the fact that it does not bind to alpha-fetoprotein (Sheehan and Branham, 1987), EE2 can affect endocrine and neuroendocrine systems, and consequently impair the ability of wildlife and humans to reproduce (National Toxicology Program, 2010).

Reproduction is controlled by the hypothalamic Gonadotropin-Releasing Hormone (GnRH) neurons (Knobil, 1988; Wierman et al., 2011). This neuroendocrine network constitutes the final output of the hypothalamus, that regulates reproduction after integrating numerous signals coming from the organism, such as circulating sex hormones, and from the environment (Herbison, 2008). The present study investigated whether developmental sub-chronic exposure to an environmental-range or a pharmacological dose of EE2 from critical fetal and perinatal periods up to puberty disturbed reproductive function in adult female mice, including physiological and behavioral parameters, and neuroendocrine networks regulating the hypothalamic-pituitary-gonadal (HPG) axis.

We already demonstrated in our laboratory that exposure to EE2 altered ontogenesis of GnRH neurons in mouse embryos, by increasing the number of these neurons (Pillon et al., 2012). In this previous study, embryos were exposed during a short period to specifically target GnRH neuron neurogenesis and nasal migration, which occur between embryonic day (E) E10 and E13 in mouse. In the current study, we investigated whether this alteration may persist into adulthood in females exposed to EE2 from fetal to peripubertal life. The neuroanatomy of the hypothalamic GnRH neuronal network was studied in adult female mice, both in the preoptic area (POA) where most of the GnRH cell bodies are scattered, and in the median eminence (ME), in which most GnRH axonal terminals are concentrated. As the highly estrogen-sensitive kisspeptin neuroendocrine network closely regulates GnRH neurons activity to control gonadotropins' secretion (Piet et al., 2013; Yeo, 2013), we also analyzed kisspeptin neurons immunoreactivity in the POA. Since alterations in such main neuroendocrine networks should impact on physiological reproductive parameters, we assessed the onset of puberty, the length of the estrus cycle and its regularity, and fertility in adult females. Moreover, perinatal and peripubertal estrogens are known to exert a facilitator role on female brain organization (Bakker et al., 2003) for the expression of maternal (Keller et al., 2010) and socio-sexual (Bakker et al., 2002) behaviors in adulthood. Thus, to test the hypothesis that EE2-exposure during development may affect these behaviors in adult females, we assessed maternal nurturing behaviors, mating partner preference, and sexual receptivity. Finally, we evaluated the anxiety level of adult females. Anxiety is known to be sensitive to estrogens and estrogen-like molecules during brain sexual differentiation (Farabollini et al., 1999; Dugard et al., 2001) and may trigger several reproductive-related behavioral disorders such as maternal care (Neumann, 2008).

## Materials and methods

### Animals

**Figure 1:**
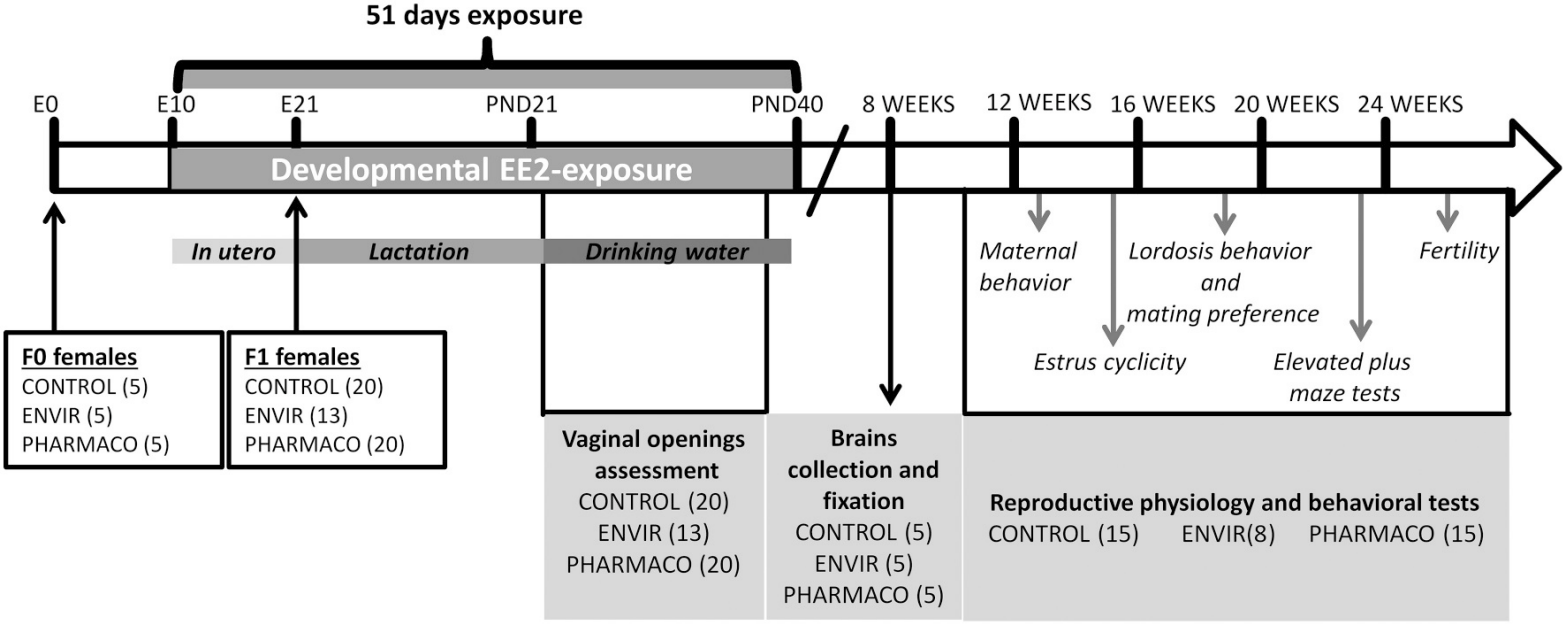
**Experimental design**. F1 females were exposed to 0.1 and 1 μg of ethinylestradiol (EE2)/kg (body weight)/day, corresponding respectively to environmentally-relevant (ENVIR) and pharmacological (PHARMACO) doses. Exposure began at embryonic day (E) 10 by exposing F0 pregnant and then lactating dams through their drinking water until postnatal day (PND) 21. After weaning, F1 females continued to be exposed through drinking water until PND40. Vaginal opening was assessed during the peripubertal period (PND23 to PND32). Neuroanatomical studies were performed on 8-week-old females. Estrus cyclicity was assessed between 14 and 17 weeks of age. Maternal behavior was assessed on nulliparous females. Social-sexual preference tests were performed on sexually-naïve females, lordosis behavior was then assessed in 7 trials which were followed by a second test of social-sexual preference. Anxiety-like behavior was assessed in an elevated plus maze device. Finally, females were mated with fertile males to assess fecundity and fertility.

All experiments were conducted in accordance with the European directive 2010/63/EU^2^ on the protection of animals used for scientific purposes (agreement number E37-175-2) and approved by an ethical committee for animal experimentation (CEEA Val-de-Loire, Tours, France, C2EA-19). Fifteen pregnant Swiss mice (F0), purchased from a commercial breeder (Charles River—France), were divided into three groups: CONTROL (*n* = 5), ENVIR (*n* = 5), and PHARMACO (*n* = 5) (see Figure 1 for Experimental design). Mice were housed in individual standard cages (45 × 25 × 15 cm^3^) and given free access to food (Safe, Augy, France) and water, under controlled temperature (22°C), humidity (50–60%) and photoperiod cycle (12 h light/12 h dark).

### Ethinylestradiol treatment

A stock solution of EE2 (Sigma Aldrich, Saint-Quentin-Fallavier, France) was prepared in 100% ethanol at 1 μg.mL^−1^. Dilutions to final doses of exposure were prepared in drinking water. The daily dose was calculated according to the animals' weights and their water consumption. Animals were exposed to EE2 from embryonic day (E) E10 through pregnant dams' (F0 generation) exposure to drinking water. After birth, pups were sexed; litters were culled to four females and four males *per* dam and returned to their mothers up to weaning. Animals (F1 generation) continued to be exposed through feeding up to weaning. After weaning, animals were separated from their mothers and from the opposite sex individuals. Females were housed in standard cages (4–5 females *per* cage) and continued to be treated with EE2-containing drinking water until postnatal day 40 (PND40) (Figure 1). The three groups were treated as follows: two groups were exposed to EE2, the ENVIR group exposed to 0.1 μg/kg body weight (bw)/d (day) (*n* = 13 females), a dose corresponding to an exposure range found in highly polluted environments (Mashchak et al., 1982), and the PHARMACO group (*n* = 20 females) exposed to a pharmacological dose of 1 μg/kg bw/d of EE2 (Stanczyk et al., 2013). The third group received vehicle without EE2 (CONTROL group; *n* = 20 females).

Among the five F0 females in the ENVIR group, only three were pregnant although vaginal plugs had been observed in the five females, leading to the effective of 13 F1 females in the ENVIR group (4 female' pups for two females and 5 female' pups for the third one), instead of 20 for the CONTROL and PHARMACO groups (4 female' pups for each FO female).

This experimental design was established to study the effect of EE2 on female and male reproductive function. Due to the different aspects being assessed requiring different scheduling and experimental procedures on animals (estrus cyclicity, behavioral tests), female and male EE2 effects were studied separately. Only the results obtained from female offspring are presented here.

### Reproductive physiology

#### Body weight

Females from the tree treatment groups were weighted from PND22 up to PND92.

#### Vaginal opening

The age at vaginal opening was assessed through daily visual examination. Vaginal opening was observed each day from PND23, until complete opening was detected on all the females.

#### Cyclicity in adult females

To evaluate the regularity of the estrus cycles, daily vaginal smears were carried out over 3 weeks between postnatal weeks 14 and 18. Smears were collected by flushing the entrance of the vagina with physiological saline, which was then colored with methylene blue to visualize the cells under optical microscopy. The stages of proestrus, estrus and metestrus/diestrus were determined from the cytology observed on smears.

#### Fertility study

Female fertility was assessed at the end of all the behavioral analyses. Each 26-week-old female was housed with a fertile male mouse for 1–14 days until a vaginal plug was observed. The number of pups and litters were recorded for each experimental group of females.

### Neuroanatomical studies

#### Tissues collection and preparation

Fifteen 8-week-old females (5 *per* group) were euthanized using an intra-peritoneal injection of a lethal dose of sodium-pentobarbital (100 mg/kg). Intracardiac perfusions with a nitrite buffer solution followed by a solution of phosphate buffer saline 0.1 M, pH 7.4 (PBS) with 4% paraformaldehyde (PBS 4% PFA) were performed. The mouse heads were dissected and brains removed and post-fixed in PBS 4% PFA for 24 h. Brains were then immersed in 20% sucrose solution in PBS for cryoprotection and stored at 4°C. Each brain was embedded in TissuTek^©^ and frozen in Isopentane at −40°C, before being sliced with a cryostat into 20 μm coronal sections collected on SuperFrost glass slides (Menzel, Germany) and stored at −20°C until immuhistochemistry.

#### Immunohistochemistry for GnRH neurons

Thirty serial coronal brain slices (Bregma 0.14 to Bregma 0.86; Franklin and Paxinos, 1997) covering within a rostro-caudal axis the median septum (MS), the *Organum Vasculum of the Stria Terminalis* (OVLT) and the medial preoptic area (mPOA) from each female were immunolabeled for GnRH perikarya. To label GnRH terminal nerves in the median eminence (ME), three coronal sections *per* female were selected from the rostral, medial and caudal ME (Bregma −1.70, Bregma −1.94, and Bregma −2.08 respectively; Franklin and Paxinos, 1997).

Brain slices were treated for 15 min at room temperature (RT) in PBS with 0.3 Triton X-100 (PBST) and 1% H_2_O_2_ to block endogenous peroxidases, and then rinsed three times (3 × 5 min). After being incubated for 1 h at RT in PBST and 10% of normal goat serum (PBST-NGS) to reduce background noise, slices were incubated in the primary antibody 19,900 rabbit IgG (1:3000) (Geller et al., 2013) diluted in PBST-NGS overnight at 4°C. For GnRH perikarya labeling, sections were rinsed three times (3 × 5 min) in PBS and incubated for 2 h at RT with the secondary biotinylated anti-rabbit immunoglobulin goat antibody (Vector Lab) diluted at 1:500 in PBST-NGS. Slices were washed twice in PBS and once in Tris-HCl buffer (0.05 N, pH 7.6), before being incubated for 1 h in ABC peroxidase (horseradish peroxidase) complex [Vector Laboratories, Burlingame, CA, USA kit Vectastain Elite (PK6100)] at a dilution of 1:600 in PBST. The signal was revealed with 3.3′′ diaminobenzidine (DAB) and 0.02% H_2_O_2_. The enzymatic reaction was stopped in Tris-HCL. Finally, sections were dehydrated in graded alcohol and toluene, and mounted with DEPEX.

GnRH terminal nerves in the ME sections were labeled by rinsing three times in PBS and then incubating in the secondary antibody goat anti-rabbit IgG Alexa 546 (Molecular Probes) diluted at 1:1000 in PBST-NGS for 2 h at RT. The secondary antibody was rinsed three times in PBS and nuclei were counterstained with DAPI (1:1000) for 1 min. Sections were washed, mounted on glass slides and coverslipped with Fluoromount-G (Southern Biotech, Birmingham, AL).

#### Analysis of GnRH cells bodies and terminal nerves

Counting of GnRH neurons perikarya was performed under a light microscope at 20X magnification. One brain from the ENVIR group presented high background noise with DAB labeling and was excluded from this analysis. As GnRH neurons perikarya are scattered in their distribution area and distinct from each other, it is easy to identify individual GnRH labeled neurons between the n and the n+1 slices according to their neuroanatomical location (Zhu et al., 2015). Immunoreactive perikarya in 30 serial slices within a rostro-caudal continuum were counted (Bregma 0.14 to Bregma 0.86; Franklin and Paxinos, 1997). The total number of summed neurons from the 30 slices *per* animal was compared between groups. Subsequently, to compare GnRH neuron distributions, the number of neurons from each five consecutive slices was summed to establish a distribution curve of neurons from the MS up to the mPOA.

Analyses of GnRH terminal nerves in the ME were performed using epifluorescence microscope images computerized with Mercator Software (Explora Nova, La Rochelle, France). Under a magnification of 20X, anatomical regions in the ME were localized using DAPI labeling. A selected region was centered in a rectangle of 10,000 μm^2^ and labeling was observed at a wavelength of 555 nm. The image was captured, digitalized and thresholded to detect the GnRH immunolabeled area. This area was automatically calculated and divided by the total surface of the region to obtain a percentage. To get a surface of labeled GnRH area, an average surface area was obtained from the three sections corresponding to rostral, middle and caudal ME for each animal. The final value of labeled area *per* group is a mean of the GnRH surface for 5 animals.

#### Immunohistochemistry for kisspeptin neurons

Two coronal 20 μm brain slices *per* female from the periventricular preoptic nucleus (PVpo) (Bregma 0.02 mm; Franklin and Paxinos, 1997) were processed to immunostain kisspeptin neurons (Clarkson et al., 2014). Slices were incubated in PBS with 0.3 Triton X-100 and 10% of normal goat serum (PBST-NGS) for 1 h at RT, and then in sheep anti-kisspeptin antibody (AC053) at a dilution of 1:2000 (Franceschini et al., 2013) at 4°C overnight. The following day, slices were rinsed three times (3 × 5 min) with PBS, and then processed for immunofluorescence labeling for 2 h at RT using Alexa 546-conjugated donkey anti-sheep IgG second antibody (1:1000; Molecular Probes). After three rinses in PBS, nuclei were counterstained with DAPI (1:1000) and incubated for 1 min. Slides were rinsed in water and coverslipped with Fluoromount-G (Southern Biotechnology, Birmingham, AL, USA), before being stored in the dark at 4°C.

### Behavioral analyses

Two weeks before starting the behavioral tests, females were housed individually in a standard cage with free access to food and water. Except for the elevated plus maze test, all the behavioral tests were conducted under red light during the dark phase of the dark/light cycle 2 h after lights off.

#### Maternal behavior

Females were tested for induction of maternal behavior by exposing them to cross-fostered newborn pups (Rosenblatt, 1967; Keller et al., 2010). Individually housed 12- to 14-week-old females were tested as nulliparous in their usual home cage after replacing the top with a clear Plexiglas cover to allow observation. Each female was allowed a 5-min habituation period before maternal behavior was assessed for 30 min. To this end, 3- to 5-day-old pups from another female were placed at the opposite sides of the cage (three *per* female). Measures recorded were: the latency to retrieve the first pup to the nest, and then the cumulative duration over the 30-min test for each of the following behaviors: sniffing pups, licking/grooming, nursing (arched-back position), nest building and self-grooming, while other activities such as rearing, leaning or digging were recorded as non-maternal care behaviors.

#### Lordosis behavior

Lordosis behavior was assessed in transparent Plexiglas aquaria during the first 2–5 h after lights off. To evaluate normal physiological response, intact-estrus females were used. Each 17- to 23-week-old female was tested seven times, once *per* estrus cycle. Each estrus female was placed in the aquarium with a stimulus male for 20 min. Stimulus males were allowed to become habituated to the aquaria with their own bedding at least 2 h before introducing the female. The number of attempted mounts, successful mounts and lordosis postures (when pelvic thrusts were observed) were scored. To avoid any unwanted pregnancies, the male was removed from the female 3 s after intromission (pelvic thrust). If females received 20 attempted or successful mounts, the test was stopped before the end of the 20-min trial. The lordosis quotient (LQ) was calculated as the number of scored lordosis postures/total number of successful mounts × 100. The first three trials served as experience acquisition for the females and the LQ was scored from the fourth to the seventh trial (Kercmar et al., 2014).

#### Social-sexual investigatory behavior

The mating preference of females was evaluated through a choice between a gonad-intact male and a castrated male. Females were tested during estrus, first as sexually inexperienced (naïve), and then after sexual interactions with males (16- and 24-week-old respectively), in a Plexiglas device divided into three compartments enabling free movement of the tested animal. Lateral compartments were divided into two and the partition had small holes at its base allowing diffusion of odors and nose-to-nose contacts. The cumulative time spent in each lateral compartment and the cumulative time spent sniffing each male were recorded for 10 min.

#### Elevated plus maze test

An elevated plus maze test (EPM) was used to assess the anxiety level of 25-week-old females. The EPM consists of two open and two closed cross-shaped arms (5 cm wide × 40 cm long) elevated 50 cm from the floor. Each diestrus female was placed in the central square and allowed to investigate the EPM arms for 5 min. The time spent in the two open and two closed arms and the number of entries into each arm were recorded.

### Statistical analyses

Statistical analyses were performed with GraphPad prism5 software (GraphPad Software San Diego, CA). Normality of distributions was tested using the D'Agostino and Pearson omnibus normality test. A Two-way ANOVA with the Bonferroni post-test was used to compare body weights across animal age, and to compare and analyze lordosis behavior data. A Chi^2^ test was used to compare the percentages of vaginal opening and lordosis postures. Time spent sniffing gonad-intact and castrated males in the sexual preference test were compared using a paired *t*-test for each group. A One-way ANOVA with the Bonferroni multiple comparison test was used to compare the three groups of animals when distributions were Gaussian and variances equal (Bartlett test) (estrus cycle length). If data did not fit Gaussian distribution and/or variances were unequal, we used a non-parametric Kruskal-Wallis with Dunn's multiple-comparison test (maternal behavior, anxiety-like behavior and number of GnRH neurons). GnRH neurons distribution was compared with an Extra sum-of-square-test F. Differences were considered significant for *p* < 0.05. Non-parametric or parametric data are respectively presented as Tukey's boxplots or as histograms (mean ± SEM).

## Results

### Reproductive physiology

#### EE2 and body growth curves

**Figure 2:**
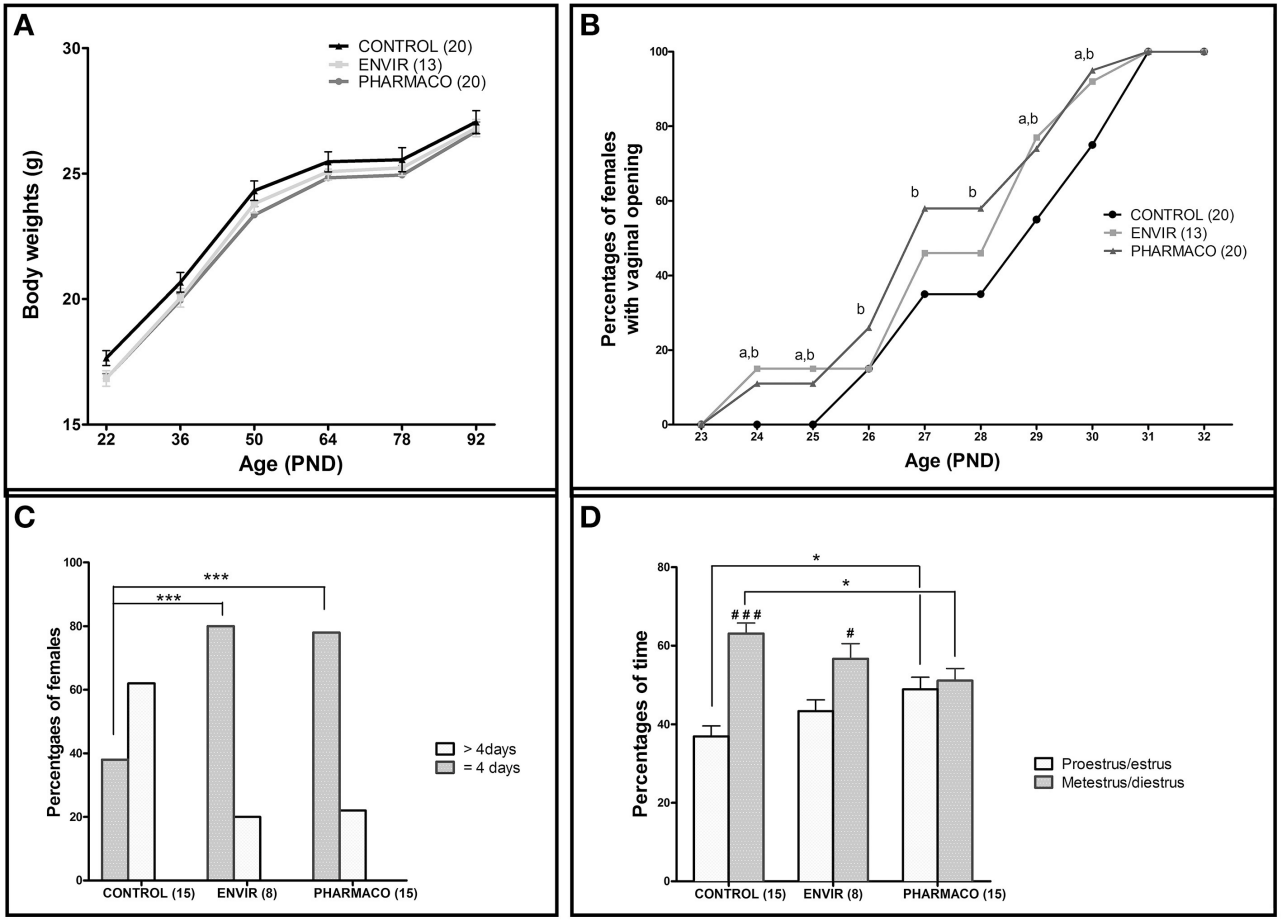
**Effects of developmental EE2 exposure on reproductive physiology**. **(A)** Body weight of female mice exposed to the vehicle (Control; *n* = 20) or to EE2 [ENVIR (*n* = 13) and PHARMACO (*n* = 20)] was evaluated from PND22 up to PND92. Data are expressed as means ± SEM. **(B)** Vaginal opening pattern for each experimental group. Cumulative percentages of female mice showing vaginal opening according to age and experimental condition are presented from PND23 to PND32. Dissimilar letters indicate significant differences at each PND, *p* < 0.05 using the Chi^2^ test of Pearson (a = ENVIR vs. Control. b = PHARMACO vs. Control. **(C,D)** The estrus cycle was evaluated daily for 14- to 18-week-old females during three cycles. **(C)** Estrus cycle length given as a percentage of females showing a complete cycle over four consecutive days (4 days) and females that did not complete a cycle over 5 days of vaginal cytology analyses (>4 days). Chi^2^ test comparing Control vs. ENVIR or Control vs. PHARMACO groups, ^***^p < 0.001. **(D)** Average occurrence of proestrus/estrus (follicular phase) vs. metestrus/diestrus (luteal phase) stages and *per* experimental group during 3 weeks of vaginal cytology analyses in adult females. One-way ANOVA (*F* = 4.32). ^*^Tukey's multiple comparison test; ^*^p < 0.05. Paired *t*-test: ^#^*p* < 0.05; ^*###*^*p* < 0.001.

Two-way ANOVA showed that the kinetics of body weight exhibited a significant overall effect of EE2-treatment [*F*_(2, 300)_ = 6.22, *p* = 0.002] and age [*F*_(5, 300)_ = 291.78, *p* < 0.0001] on growth curve between PND22 and PND92. In spite of a statistically significant effect of EE2-treatment, this accounts for approximately 0.68% of the total variance against 80% for age-effect. Bonferroni post-test did not show any statistically significant effect of EE2 for both ENVIR and PHARMACO doses at different ages (Figure 2A).

#### EE2 and vaginal opening

Vaginal opening was detected from PND24 in 15 and 10% of the EE2-exposed females in the ENVIR and PHARMACO groups respectively, whereas no vaginal opening was detected in CONTROL females (Figure 2B). A Chi^2^ test revealed significant earlier vaginal opening in EE2-exposed animals in PND24 and PND25 (*p* = 0.0007). In CONTROL females, the first vaginal openings were detected at PND26 (Chi^2^ CONTROL vs. PHARMACO, *p* = 0.05). Then, up to PND31 when all the females of the three groups presented an opened vagina, the proportion of females with vaginal opening was always statistically higher in the EE2-exposed females than in the unexposed CONTROL females. The Chi^2^ test revealed significant differences in the cumulative percentages of female mice with vaginal opening between the three experimental groups from PND24 to PND31.

#### EE2 and estrus cyclicity

The estrus cyclicity of the adult females was assessed through daily vaginal smears for a period of three estrous cycles beginning at the 14th week of age. Histograms in Figure 2C show the percentages of females which have completed a full cycle in four successive days, i.e., females showing a return to the state of the first day of the cycle on day 5 of daily vaginal smears. In the CONTROL group, only 38% of females had accomplished a complete cycle within 4 days, compared to 80 and 78% of ENVIR and PHARMACO females respectively (*p* < 0.0001). Thus, EE2-exposed females showed shortened estrus cycles.

Analyses of follicular and luteal phases' length show that, for CONTROL females, 37% of the total cycle length was proestrus/estrus, whereas for EE2-exposed ENVIR and PHARMACO females proestrus/estrus represented about 43 and 49% of the total cycle length respectively [One-way ANOVA, *F*_(2, 42)_ = 4.32, *p* = 0.01; Figure 2D). The Bonferroni multiple comparison test showed that the lengths of the follicular and luteal phases were statistically significantly different from the CONTROL females only for the PHARMACO group (*p* < 0.05). The intragroup comparison of the cycle phases' lengths showed that, in CONTROL females, the proestrus/estrus phase is highly significantly shorter (37%) than the metestrus/diestrus phase (63%) (paired *t*-test; ^*###*^*p* < 0.001). Within the ENVIR group, the difference between the two phases was also significant (43% in proestrus/estrus and 57% in metestrus/diestrus; ^#^*p* < 0.05), as it was not in PHARMACO females which spent 49 and 51% in proestrus/estrus and metestrus/diestrus respectively.

#### EE2 and fertility

No differences in fecundity and fertility were detected, since the relative number of litters (100% CONTROL, ENVIR, and PHARMACO mated females farrowed) and litter size (11.3 ± 0.8, 12.4 ± 1.1, and 10.5 ± 1.3 in CONTROL, ENVIR, and PHARMACO respectively) did not vary. There was no difference in sex ratio (number of males/number of females) between the three groups: 0.96 ± 0.18, 1.21 ± 0.17, and 1.16 ± 0.26 in CONTROL, ENVIR, and PHARMACO groups respectively (Kruskal-Wallis test, *p* = 0.70; data not shown).

### Neuroanatomical studies

#### EE2 and the neuroendocrine GnRH system

**Figure 3:**
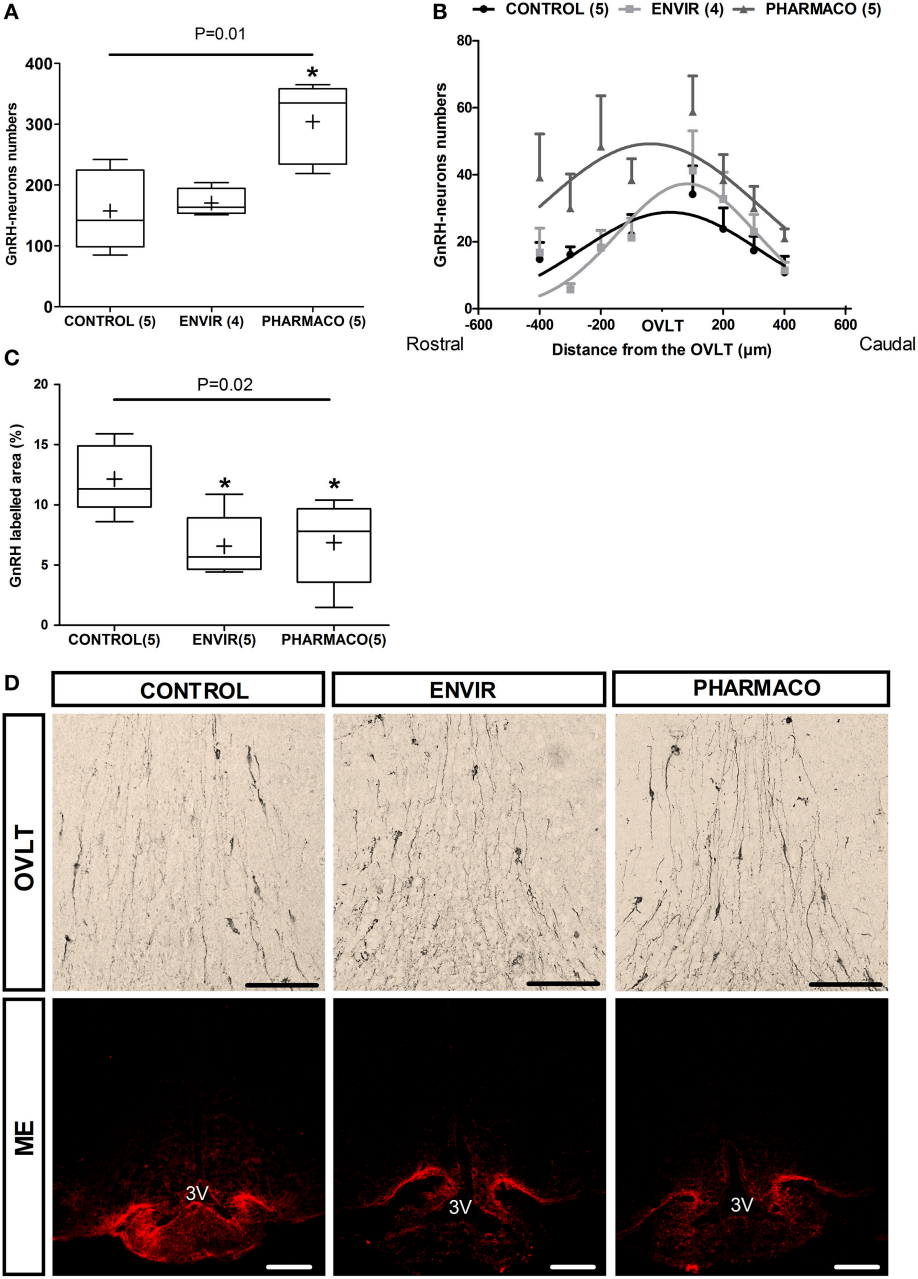
**EE2 altered the establishment of GnRH neuron network**. **(A)**Total number of GnRH immunoreactive neurons in the POA for each experimental group. **(B)** Curve fit of the distribution of GnRH neurons centered on the OVLT through a rostro-caudal axis from the median septum (-400 μm) up to the medial preoptic area mPOA (+400 μm). Extra sum-of-square-test *F*_(6, 103)_ = 5.75, *P* < 0.0001. **(C)** Quantification of the labeled area of GnRH in three parts of the median eminence (posterior, middle and anterior). Tukey's box-plots of GnRH labeling according to the experimental group. The band is the median and (+) is the mean. Kruskal-Wallis test; ^*^p < 0.05. Dunn's Multiple Comparison test (statistically different from Control). Numbers in brackets are effectives of animals *per* group. **(D)** Representative photographs of immunohistochemical labeling with DAB-Ni staining of GnRH perikarya in the *Organum Vasculum* of the *Lamina Terminalis* (OVLT) (upper panel) and immunohistochemical staining with fluorescence of GnRH fibers in the median eminence (ME) (lower panel). 3V: third ventricle. Scale bar = 100 μm.

The effects of developmental exposure to EE2 on the numbers and neuroanatomical distribution of hypothalamic GnRH neurons perikarya in adult female mice were assessed (Figure 3). Immunohistochemical analysis of the GnRH neuroendocrine network in the main areas of their perikarya distribution in the median septum (MS), the *Organum Vasculum* of the *Lamina Terminalis* (OVLT) and the medial preoptic area (mPOA) revealed a statistically significant effect of EE2 treatment on the number of neurons (Kruskal-Wallis test, *p* = 0.01) (Figure 3A). Dunn's multiple comparison test revealed a significant increase in females exposed to the PHARMACO dose during development, with an increase to 193% (304 ± 29 neurons) compared with the CONTROL group (158 ± 30 neurons). No significant difference was detected for the ENVIR group (171 ± 11 neurons) compared to the CONTROL group. Figure 3D (upper panel) illustrates these differences in GnRH neuron numbers in the OVLT between the three groups.

The neuroanatomical distribution of the GnRH neurons was investigated through a rostro-caudal axis within a continuum centered on the OVLT and extending from the MS (-400 μm) up to the mPOA (+400 μm) (Figure 3B). The number of neurons is indicated in each 100 μm position by summing the number of neurons from five serial slices. In CONTROL females, the distribution shows a bell-shaped curve whose peak is located at the OVLT level. Statistical comparison using an Extra sum-of-square-test F shows statistically significant differences between the three curves [*F*_(6, 103)_ = 5.75, *p* < 0.0001], indicating that neuronal distribution in adult has been disturbed by EE2 developmental exposure (Figure 3B).

Most of the hypothalamic GnRH neurons project their axons into the ME. The analysis of the anterior, middle and posterior parts of the ME from each animal allowed the proportion of GnRH labeled area to be determined, reflecting the density of GnRH fibers. In females that were developmentally exposed to EE2 (ENVIR and PHARMACO groups), the GnRH labeled area was significantly reduced. Results are shown in Figure 3C, illustrated in Figure 3D microphotographs (lower panel). Compared to the CONTROL group (12.1 ± 1.3%), the reduction was about 46% in the ENVIR group (6.6 ± 1.2%), and 44% in the PHARMACO group (6.9 ± 1.5%) (Kruskal-Wallis test, *p* = 0.02; Dunn's multiple comparison test, ^*^p < 0.05).

#### EE2 and kisspeptin neurons

**Figure 4:**
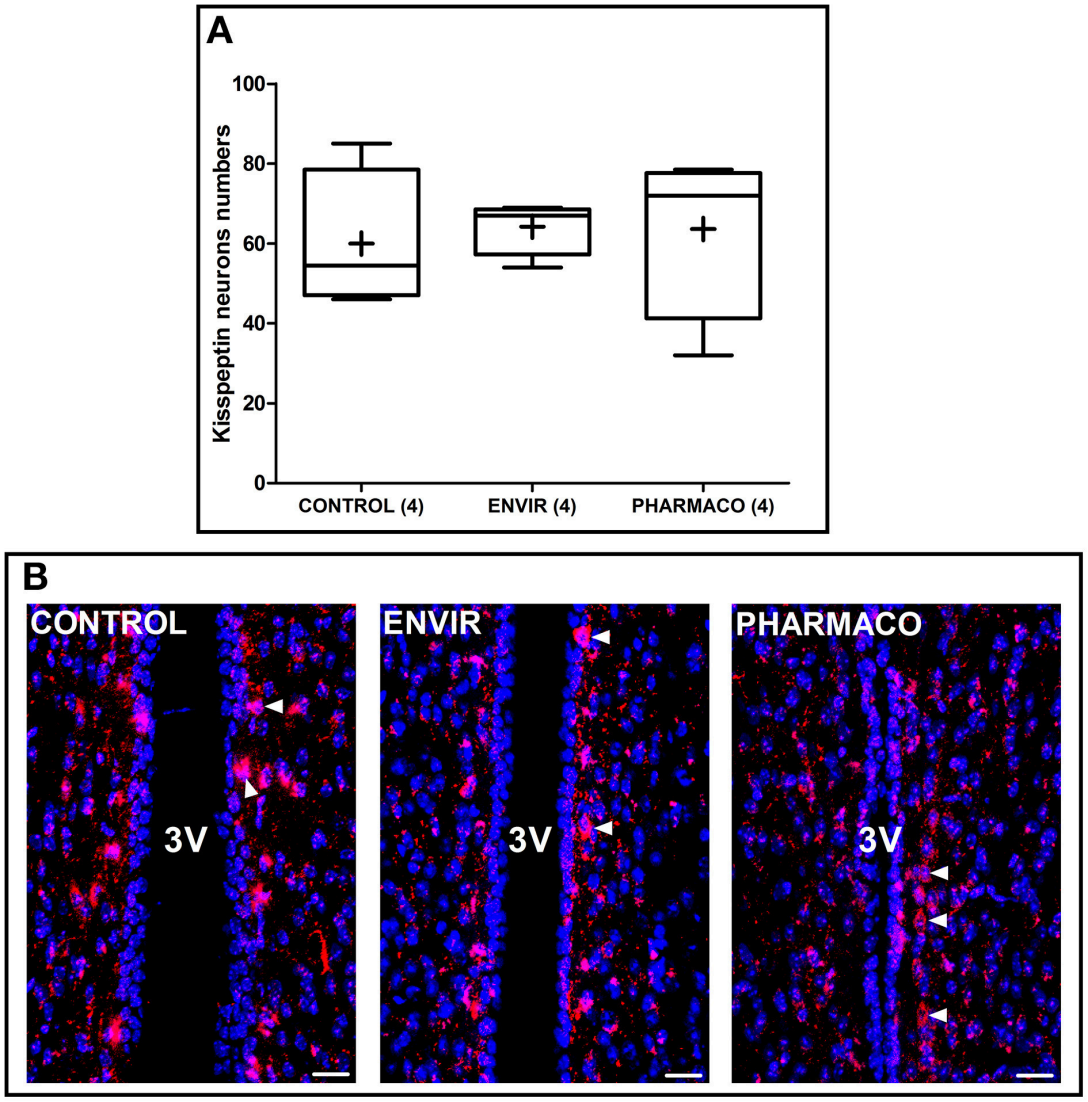
**The number of kisspeptin neurons was not disturbed by EE2 exposure**. **(A)** Tukey's boxplots of the numbers of kisspeptin neurons counted in the periventricular preoptic nucleus (PVpo). (+) is the mean, numbers in brackets are effective of animals *per* group. No statistical differences were detected. **(B)** Photomicrographs of coronal brain sections counterstained with DAPI (blue) and immunostained for kisspeptin (red). Arrowheads indicate kisspeptin perikarya along the third ventricle (3V). Scale bar = 20 μm.

Kisspeptin neurons were counted in two adjacent coronal slices in the periventricular preoptic nucleus (PVpo) for each animal. Figure 4A represents Tukey's boxplots of the mean numbers in the two slices for each animal of the three groups. As illustrated in Figure 4B, no difference *per* slice *per* animal was detected between CONTROL and EE2-exposed females, with 60 ± 9, 64 ± 3, and 64 ± 11 neurons in the CONTROL, ENVIR, and PHARMACO groups respectively.

### Behavioral analyses

#### EE2 and maternal behavior

Females of the ENVIR group spent significantly more time (980 ± 206 s) to retrieve the first pup to the nest than CONTROL and PHARMACO females (543 ± 159 s and 673 ± 152 s respectively) (Kruskal-Wallis test, *p* = 0.05; Dunn's multiple comparison test, *p* < 0.05) (Table 1). EE2-treated females spent significantly more time in activities that are not pups-related (51.28 ± 7.78% and 41.69 ± 5.87% in the ENVIR and PHARMACO groups, respectively), compared to the CONTROL group (29.07 ± 6.44) (Kruskal-Wallis test, *p* = 0.04). No statistically significant effect was observed for any of the other evaluated parameters related to pups' nursing (Table 1).

**Table 1:** **Maternal behavior tests**.

**Experimental Group Scored parameter**	**Control (15)**	**ENVIR (8)**	**PHARMACO (15)**
Latency to retrieve the first pup to the nest	543±159s	980±206^*^s	673±152s
Sniffing	3.35±0.70	4.20±0.87	3.93±0.60
Licking/grooming	29.13±3.5	22.57±3.77	18.41±3.30
Arched-back nursing	23.28±5.12	8.30±3.52^a^	15.28±3.84
Nest building	9.94±1.42	7.08±1.04	11.51±2.46
Self-grooming	5.44±1.28	6.56±1.65	9.19±1.73
Not pups-related activities	29.07±6.44	51.28±7.78^*^	41.69±5.87

*The latency to retrieve the first pup to the nest is shown in seconds (mean ± SEM). Results are mean percentages (±SEM) of time spent per animal in each scored behavior during a 30-min test*.

* Kruskal Wallis test with Bonferroni multiple comparison (p < 0.05;

a *p = 0.06)*.

#### EE2 and mate preference

**Figure 5:**
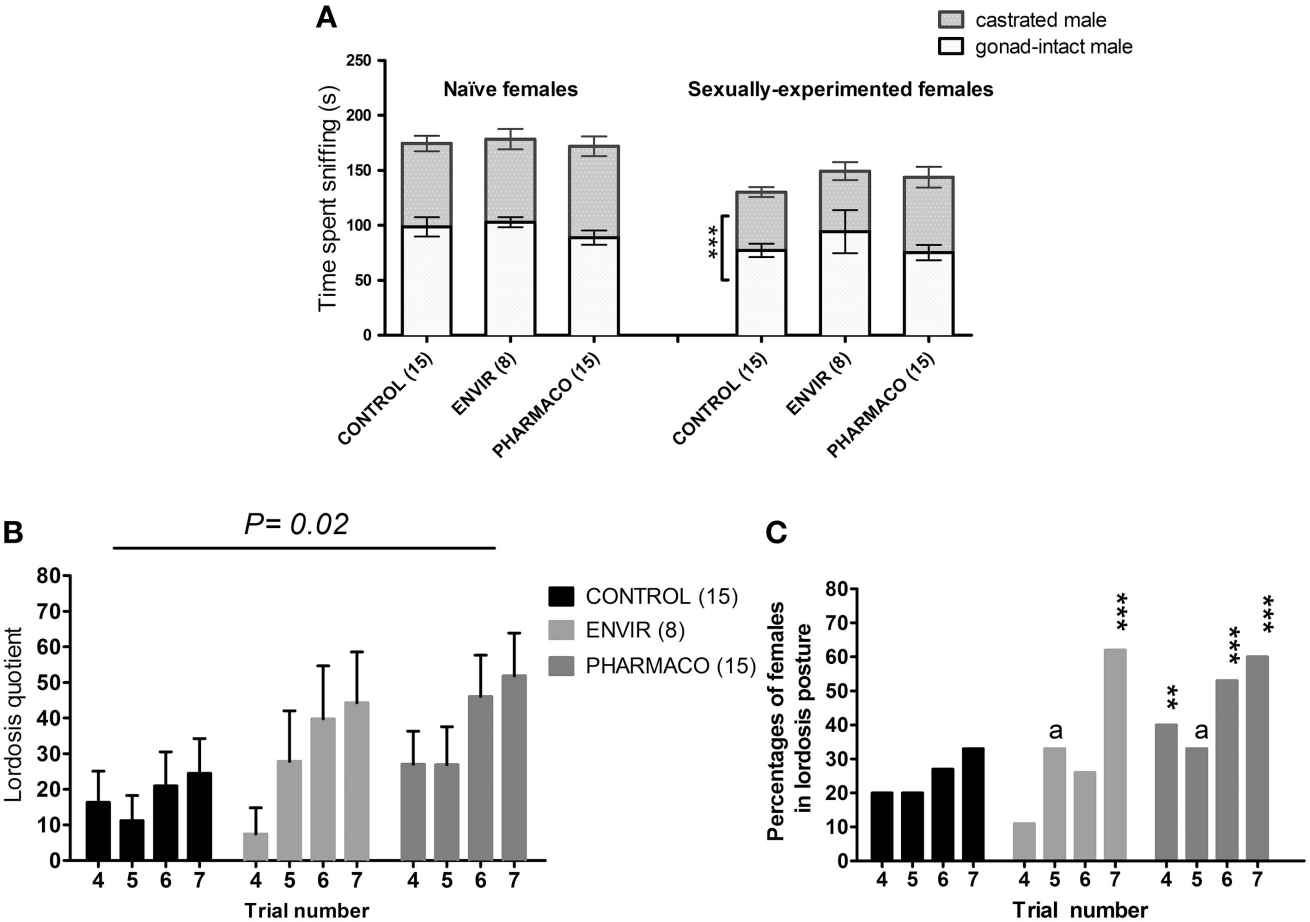
**EE2 impaired social and sexual behaviors of females**. **(A)** Sexual preference tested in naïve (without any contact with an adult male) and experienced (after mating behavior tests) females at the estrus stage. Histograms present the time spent sniffing the gonad-intact or the castrated male over 10 min. ^***^ Unpaired *t*-test comparing the average time spent by females with the gonad-intact or the castrated male. **(B,C)** Female sexual behavior (lordosis behavior): females were tested on 7 trials at the day of estrus: for each female, a trial was carried out once *per* cycle. **(B)** Lordosis quotient (LQ): number of lordosis postures/total number of successful mounts × 100. Two-way ANOVA, *p* = 0.02. **(C)** Percentages of females showing lordosis posture during behavioral tests. Chi^2^ test comparing each exposed group to the Control group for each trial. ^*a*^*p* = 0.06; ^**^p < 0.01; ^***^p < 0.001.

Mating preference tests on naïve females showed that females from the three groups did not exhibit any preference for the gonad-intact or the castrated male (Figure 5A). In contrast, the sexually-experimented CONTROL females exhibited a preference for the gonad-intact male over the castrated male (*t*-test, *t* = 4.29; *p* = 0.009). This preference was found neither in ENVIR females (*t*-test, *t* = 1.67; *p* = 0.14) nor in PHARMACO females (*t*-test, *t* = 1.02; *p* = 0.3).

#### EE2 and lordosis behavior

A Two-way ANOVA test demonstrated an overall significant effect of trial number [*F*_(3, 143)_ = 2.95, *p* = 0.03] and EE2-exposure on the lordosis quotient (LQ) [*F*_(2, 143)_ = 3.94, *p* = 0.02]. As an example, during the seventh trial, PHARMACO females showed a 200% increase in the LQ compared with the CONTROL females (Figure 5B).

The percentages of females showing a lordosis posture were also compared between the three different groups (Figure 5C). During the fourth trial, 20% of the CONTROL females showed a lordosis posture, vs. 11 and 33% of females in the ENVIR and PHARMACO groups respectively. This rate increased to 33% during the seventh trial for the CONTROL group vs. 63 and 60% for the ENVIR and PHARMACO groups respectively. A Chi^2^ test revealed a statistically significant increase (*p* = 0.0002) for the ENVIR and PHARMACO groups (Figure 5C).

#### EE2 and anxiety-like behavior

**Figure 6:**
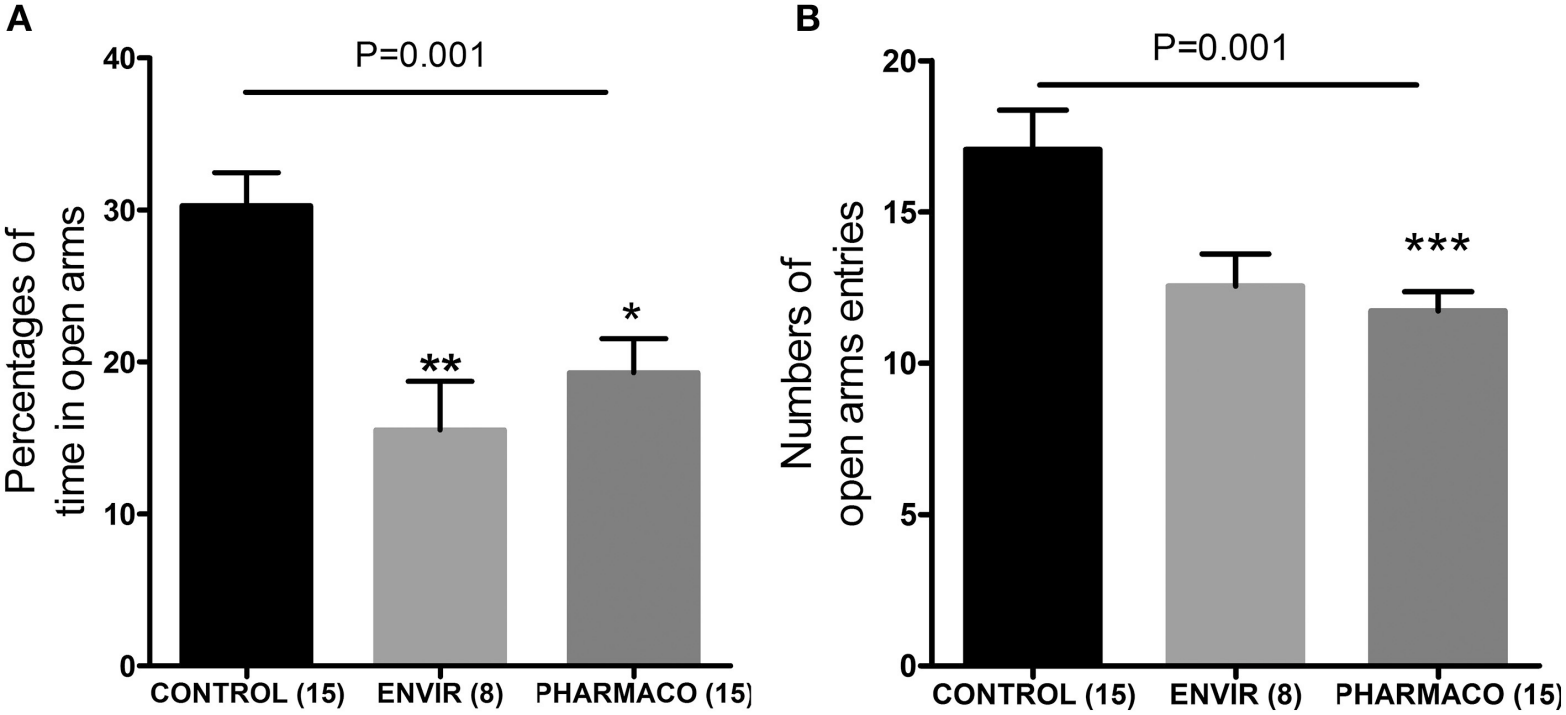
**EE2-exposure increased anxiety of females**. **(A)** Percentages of time spent in the open arms of an elevated plus maze (EPM) (Kruskal Wallis test; *p* = 0.001). **(B)** Numbers of entries into the open arms of the EPM (Kruskal Wallis test; *p* = 0.01). Bonferroni multiple comparison test: ^*^p < 0.05; ^*^p < 0.001; ^***^p < 0.001.

The EPM test revealed that EE2 treatment increased the anxiety-like behavior of the F1 generation females in adulthood. Females from the ENVIR and PHARMACO groups spent less time in the open arms of the EPM device than the CONTROL group (Kruskal-Wallis, *p* = 0.01, with ^*^Bonferroni multiple comparison; *p* < 0.001) (Figure 6A). EE2-exposure also decreased significantly the number of entries into the open arms from 17.1 ± 1.3 in the CONTROL group to 12.6 ± 1.1 in the ENVIR group (*p* < 0.001) and 11.7 ± 0.6 in the PHARMACO group (Kruskal-Wallis, *p* = 0.001; with ^*^Bonferroni multiple comparison) (Figure 6B).

## Discussion

The environmental EE2 contamination may be considered as an emerging issue that might affect animal and human health^1^ (Owen and Jobling, 2012; Green et al., 2013). In the present study, we investigated the long-term effects of a developmental exposure to EE2 on reproductive function in female mice. We demonstrated that chronic exposure to EE2 in drinking water during critical phases from intrauterine development until puberty, at doses in a range of environmental exposure and much lower than the previously reported Low-Observed-Adverse-Effect-Level (LOAEL) dose (Kanno et al., 2001), induced enduring effects on reproductive function in females. Indeed, we observed advanced vaginal opening and shortening of estrous cyclicity. The GnRH neuroendocrine network, the main regulator of the gonadotropic axis, was altered, suggesting that neuroendocrine effects are involved. Moreover, behavioral outcomes were disrupted, namely a decrease in maternal nurturing behavior, a decrease in social-sexual preference, an increased lordosis behavior and an increased anxiety level. All these points will be discussed in the following paragraphs.

### Developmental exposure to EE2 altered reproductive physiology

Our results showed that EE2 developmental exposure induced advanced vaginal opening and modifications of estrus cyclicity at both ENVIR and PHARMACO doses. Fertility and fecundity over one breeding period were not significantly affected by EE2 treatments.

We demonstrated that both doses of EE2 (0.1 and 1 μg/kg bw/d) significantly accelerated the vaginal opening when compared with the CONTROL group. These results are broadly consistent with several other studies in Sprague-Dawley rats conducted at the National Center for Toxicological Research for the National Toxicology Program (NTP) (National Toxicology Program, 2010). In these studies, authors used rats treated chronically with an oral dose range of 0.2–5.8 μg/kg/day during development up to PND140, using an experimental design comparable to our treatment protocol, even though doses were notably higher than those used herein. Likewise, they found that EE2 disturbed vaginal opening and estrus cycles at 5.8 μg/kg/d, but had no effect on fertility. Another study conducted with Long-Evans rats (Ryan et al., 2010) showed that 5 μg/kg/d of EE2 exposure from embryonic day 7 up to PND18 accelerated vaginal opening, reduced body weight at vaginal opening and induced some genital malformations in females.

In our study, EE2 exposure extended from gestation up to PND40. As females were still exposed to EE2 at vaginal opening, then the significant alteration of pubertal timing may be the consequence of peripheral effects through a direct action of EE2 on vagina epithelium, suggesting that the observed advanced vaginal opening do not necessarily imply central and/or ovarian pubertal changes. Nevertheless, the advanced vaginal opening may also result from central effects through an interference of EE2 with the neuroendocrine system during critical developmental periods (Franssen et al., 2014). Indeed, we found in adult females developmentally exposed to EE2 an alteration in GnRH neurons neuroanatomical localization in the POA and a decrease in their terminal nerves density in the ME. During embryonic development, GnRH neurons migrate from the olfactory placode through the nose up to their final main localization in the median septum, rostral hypothalamus and preoptic area (POA) (Schwanzel-Fukuda and Pfaff, 1989; Wray et al., 1989). Delay in nasal to brain migration of GnRH neurons has been associated with a delay in puberty and disruption of estrus (Parkash et al., 2012), implying that the onset of puberty is dependent on the correct timing of GnRH neuron migration. Moreover, Rasier et al. (2007) demonstrated that transient exposure to EDCs (estradiol, *o, p*′-DDT) in early postnatal life can induce an early maturation of the pulsatile GnRH secretion and a subsequent early developmental reduction of LH response to GnRH, constituting a possible mechanism of the sexual precocity.

We also demonstrated that exposure to EE2 induced shortening of estrous cycles in the two treated groups, as well as a proportional shortening metestrus/diestrus phase toward the proestrus/estrus phase, all the more in the PHARMACO group. The hypothalamic control of ovulation and estrous cycling is developmentally organized (Foster et al., 2006) and the activity of the GnRH secretion system is sexually differentiated during the postnatal period. Therefore, exposure to some EDCs can cause inappropriate sexual differentiation of the female hypothalamus and alterations in estrous cyclicity after puberty (Gore, 2008). We can suspect that the alterations that we detected in GnRH network led to disruptions in patterns of pituitary gonadotropins secretion, and consequently to altered production and secretion of ovarian hormones regulating estrus cycle. Subsequently, it will be interesting to investigate whether gonadotropic hormonal levels are altered in EE2-exposed females.

### Developmental exposure to EE2 altered the establishment of GnRH neuron network

The present study showed that EE2 exposure during a period in which GnRH neurons were developing led to an increase in the number of these neurons mainly in the POA. A previous study published by our laboratory already demonstrated that a short exposure to EE2 during the neurogenesis and the nasal migration of GnRH neurons between E10.5 and E13.5 led to a significant increase in the number of these neurons in embryos (Pillon et al., 2012). We now demonstrated that this increase in GnRH neurons number in embryos is still detectable in adult females, suggesting a developmental embryonic origin of this phenotype at adulthood. Such an increase in the number of GnRH neurons has already been reported after developmental EE2-exposure in zebrafish (Vosges et al., 2012).

In a birth-date study using bromodesoxyuridine pulse labeling during early GnRH neurons ontogeny, Jasoni et al. (2009) described that early-born GnRH neurons stopped their migration in the most rostral areas, while later-born GnRH neurons settled more caudally. In our study, in spite of the absence of difference in GnRH neurons number between ENVIR and CONTROL females, we detected a greater proportion of GnRH neurons located at the OVLT level and more caudally in ENVIR females than in the CONTROL group. This mislocalization may result from defects in the neuronal migration occurring between E11 and E16 (Schwanzel-Fukuda and Pfaff, 1989; Parkash et al., 2012).

Furthermore, we observed a dramatic decrease in the immunoreactivity of the GnRH terminal nerves for the ENVIR and PHARMACO groups compared with the CONTROL group in the ME. This difference in fibers immunofluorescence density can be due to a developmental alteration disrupting the axonal GnRH growth for projections, leading to a lowest density of GnRH fibers in this brain area. Other studies using an *in vitro* GT1-7 cell line reported that estrogenic EDCs impaired *GnRH-1* gene expression, cell survival and neurite outgrowth (Gore, 2001). It can also be due to an increased exocytotic release of GnRH detected as neuropeptide depletion within the neurons, a hypothesis compatible with the modification in estrus cyclicity that we observed in our current study. Overall, these results clearly show that the formation of the GnRH neuroendocrine network is vulnerable to estrogenic EDCs, leading to potential alterations in adult reproductive function, such as puberty onset or estrus cyclicity.

The mechanisms of action by which EE2 may affect GnRH neuron development remain unknown. GnRH neurons are usually described as not being directly affected physiologically by estrogens. However, GnRH neurons express low levels of *Esr2* gene coding for estrogen receptor β (ERβ) early in development (Skynner et al., 1999; Sharifi et al., 2002), consistent with a possible direct regulation of EE2 on GnRH neurons. Nonetheless, other mechanisms might occur, such as an indirect effect through the glial microenvironment believed to be a target of estrogenic compounds *via* ERs and GPR30 during development (McCarthy et al., 2002; Merlo et al., 2007; Rao and Sikdar, 2007) and closely communicating with developing and adult neurons (Kuo et al., 2010; Geller et al., 2013).

We could have hypothesized that the hypothalamic circuits governing the release of GnRH should be impaired. Kisspeptin neurons are highly sensitive to estrogens and kisspeptin neuronal network plays a key role in the cellular basis for estrogen feedback action on GnRH neurons in female reproductive function, including regulation of ovulation and estrous cyclicity (D'anglemont De Tassigny and Colledge, 2010; Roa et al., 2011). We did not observe any significant differences in the number of neurons within the dimorphic kisspeptin neurons population in the periventricular preoptic nucleus (PVpo).

The embryonic development of kisspeptin neurons in rodents shows no sexual dimorphism (Desroziers et al., 2012). However, it has been clearly established that the kisspeptin neuroendocrine network is highly dependent on gonadal steroids during postnatal development (Clarkson et al., 2009a), and there is some growing evidence for the susceptibility of the kisspeptin neuroendocrine network to environmental pollution during the postnatal period (Franceschini and Desroziers, 2013). In female rats, perinatal exposure to 5, 15, or 50 μg/kg/d EE2 did not induce significant changes in *kiss-1* mRNA levels in adulthood (Overgaard et al., 2013), as Takahashi et al. (2014) found that a single injection of a low dose of 0.02 μg/kg EE2 at PND1 decreased hypothalamic *Kiss-1* mRNA levels at PND14. In the present study, kisspeptin immunoreactivity was investigated in the PVpo, but not in the anteroventral periventricular (AVPV) nucleus or arcuate nucleus (Clarkson et al., 2009b). Therefore, though no change in kisspeptin neurons in the PVpo was observed, we cannot rule out an alteration of the dimorphic AVPV population or in the density of kisspeptin fibers.

### Developmental EE2 exposure decreased maternal behavior

Females were tested as nulliparous for innate maternal care on cross-fostered pups. Results showed that EE2-exposed females exhibited a lower motivation to retrieve pups and a lower level of care toward pups. Estrogenic EDCs exposure has already been shown to alter maternal behavior. Indeed, developmental exposure to the estrogenic compound Bisphenol A (BPA) has previously been shown to alter maternal behavior of females exposed as a fetus or during their own pregnancy (Palanza et al., 2002). A recent study reported that diethylstilbestrol (DES) exposure during pregnancy modified maternal behavior of females and induced higher anxiety levels in adult offspring exposed during their prenatal development and receiving care from exposed or oil-treated mothers (Tomihara et al., 2015). This latest study showed two possible effects of perinatal exposure to disrupting events. The first one is a direct effect of exposure on neural circuits leading to behavioral alteration. The second one is an indirect effect through a disruption of the dams nurturing behavior or anxiety level, which may lead to offspring's behavioral alterations. In our study, the EE2-exposed female mice demonstrated higher anxiety in an elevated plus maze test, as previously reported (Ryan and Vandenbergh, 2006; Ryan et al., 2010). Therefore, disrupted maternal behavior observed in EE2-exposed females may be attributed to a direct effect of EE2 on target specific genes or central circuits such as POA, directly involved in maternal care behaviors. Nevertheless, we cannot rule out that an altered F0 maternal care could have influenced the behavioral outcomes of their offspring behaviors at adulthood, such as maternal care and anxiety level (Tomihara et al., 2015).

### Developmental exposure to EE2 altered socio-sexual recognition and increased sexual behavior

We demonstrated that EE2 treatment modified sexual behavior of females by increasing their lordosis quotient (LQ). In rats, Ryan et al. (2010) found that EE2 treatment at doses equivalent to ours (0.15 and 1.5 μg/kg/d) did not change the LQ, as a higher dose (15 μg/kg/d) induced a significantly lower LQ. In their study, the LQ was monitored in ovariectomized (OVX) and estradiol-primed females. In our study, we recorded LQs in intact cycling females in the estrus phase to be in physiological conditions. This difference in the testing protocol could explain the apparently contrasting responses. Increased sexual receptivity after developmental exposure to BPA has been reported in intact cycling female rats (Farabollini et al., 2002) and in C57/Bl6 strain female mice (Naulé et al., 2014). Social and sexual behaviors in rodents are highly gender stereotyped as a consequence of brain sexualization occurring under steroid hormone action during perinatal organizational and peripubertal activational periods (McCarthy, 2008). Sexual differentiation of the female brain has long been considered as a default state of development in the absence of testosterone, suggesting that estrogen does not play any feminizing role (Gorski, 1985). Recently, several mouse knock-out (KO) models have provided evidence for a possible active role of estrogens in female brain feminization (Bakker et al., 2003; Brock and Bakker, 2011). Brock et al. (2011) demonstrated that aromatase KO-mice, unable to convert testosterone to estradiol, exhibited deficient lordosis behavior. In these mice, peripubertal priming with estradiol restored lordosis behavior in adulthood, thus suggesting a role of estradiol during the peripubertal period to express typical female sexual behaviors (Brock et al., 2011). Therefore, this raises the question as to whether EE2 exposure induced an organizational effect during perinatal and/or peripubertal sensitive periods leading to a “hyperfeminizing” phenotype.

A role of estrogens in the implementation of sexually olfactory cues in females during early development has been already evidenced (Pierman et al., 2008). Here we tested the females for their social-sexual preference through a choice between gonad-intact and castrated males. Our results showed that sexually experienced but not sexually naïve CONTROL females preferred the gonad-intact to the castrated male. By contrast, EE2-exposed females did not exhibit any preference, even when having experienced sexual behavior. Mating partner recognition has been reported to be less dependent on perinatal than on postnatal estrogens (Bakker et al., 2007). Partner recognition for mating behavior involves volatile and non-volatile odors detected by the main and the accessory olfactory bulbs (MOB, AOB) (Keller et al., 2006a). In the literature, partner recognition has been described as being mainly supported by MOB detection, whereas lordosis behavior involves AOB detection (Keller et al., 2006b). More research is needed to investigate whether a failure of EE2-exposed females to recognize adequate males is due to a disruption in the treatment of olfactory sensory cues involved in social and sexual interactions. Further, other downstream estrogen sensitive neural networks involved in processing of olfactory cues stimuli such as the sexually dimorphic medial preoptic area could be targeted by EE2 (Henley et al., 2011).

## Conclusion

In its report in answer to the state-of-the-science regarding the study of endocrine disruptors (EDs), the World Health Organization has provided recommendations and key concerns that policy-makers should take into account. This report highlighted the subtle effects of exposure during sensitive periods in intra-uterine, perinatal life, juvenile, and peripubertal periods, which may be observed throughout or only in later life, particularly for reproductive function. The present study is an additional piece of evidence further supporting the concept of “developmental origins of health and diseases” and providing evidence of neuroendocrine mechanisms underlying disruption. Since the neuroendocrine system regulates many physiological functions, it is not surprising that deregulation of this system could be the cause of disruption of several physiological functions such as reproductive and hormonal systems, growth and metabolic homeostasis. Therefore, considering the high sensitivity of neuroendocrine circuits and sexually dimorphic behaviors, it is essential to consider these parameters in the assessment of a disruptive potential of chemicals on animal and human health.

## Author contributions

DP, LD, MK, MM carried out the experiments. MK, DP, AD supervised the experiments. DP, LD, AD analyzed and interpreted the data. LD, AD, DP wrote the draft. MK, MM critically revised the draft. All authors approved the final version of the submitted manuscript.

## Funding

This work was supported by the French National Research Agency (ANR) grant NEED (#AF08CES 011).

### Conflict of interest statement

The authors declare that the research was conducted in the absence of any commercial or financial relationships that could be construed as a potential conflict of interest.

## Footnotes

^1^http://eur-lex.europa.eu/legal-content/EN/ALL/?uri=CELEX:32013L0039.

^2^http://eur-lex.europa.eu/legal-content/FR/TXT/?uri=CELEX:32010L0063.
